# Optogenetic Activation of A11 Region Increases Motor Activity

**DOI:** 10.3389/fncir.2018.00086

**Published:** 2018-10-11

**Authors:** Kathrin Koblinger, Céline Jean-Xavier, Sandeep Sharma, Tamás Füzesi, Leanne Young, Shane E. A. Eaton, Charlie Hong Ting Kwok, Jaideep Singh Bains, Patrick J. Whelan

**Affiliations:** ^1^Hotchkiss Brain Institute, University of Calgary, Calgary, AB, Canada; ^2^Department of Comparative Biology and Experimental Medicine, University of Calgary, Calgary, AB, Canada; ^3^Department of Physiology and Pharmacology, University of Calgary, Calgary, AB, Canada

**Keywords:** spinal cord, dopamine, descending, motor activity, locomotion control

## Abstract

Limbic brain regions drive goal-directed behaviors. These behaviors often require dynamic motor responses, but the functional connectome of limbic structures in the diencephalon that control locomotion is not well known. The A11 region, within the posterior diencephalon has been postulated to contribute to motor function and control of pain. Here we show that the A11 region initiates movement. Photostimulation of channelrhodopsin 2 (ChR2) transfected neurons in A11 slice preparations showed that neurons could follow stimulation at frequencies of 20 Hz. Our data show that photostimulation of ChR2 transfected neurons in the A11 region enhances motor activity often leading to locomotion. Using vGluT2-reporter and vGAT-reporter mice we show that the A11 tyrosine hydroxylase positive (TH) dopaminergic neurons are vGluT2 and vGAT negative. We find that in addition to dopaminergic neurons within the A11 region, there is another neuronal subtype which expresses the monoenzymatic aromatic L-amino acid decarboxylase (AADC), but not TH, a key enzyme involved in the synthesis of catecholamines including dopamine. This monoaminergic-based motor circuit may be involved in the control of motor behavior as part of a broader diencephalic motor region.

## Introduction

Activation of the posterior hypothalamus, zona incerta or lateral hypothalamus can elicit a variety of motor behaviors, including locomotion (Shik and Orlovsky, 1976; Sinnamon et al., 1984; Sławińska and Kasicki, 1995; Young et al., 2009). A general finding was that the activity evoked from these areas was more complicated than the one elicited from motor regions of the brainstem (Mori et al., 1989), including the mesencephalic locomotor region and the medullary reticular formation (Kim et al., 2017). For example, stimulation of the lateral hypothalamus can result in varied responses ranging from feeding to predatory attack depending on context (Stuber and Wise, 2016; Kim et al., 2017). In this work, we focus on targeting diencephalic areas of the brain containing monoaminergic cells, with the goal of understanding their multifaceted motor function.

Dahlström and Fuxe (1964) identified a series of dopaminergic nuclei named A8-16. Some of these are well linked with motor functions such as the substantia nigra pars compacta (A9), while the role of others have been less well described. One of these nuclei is the A11 region which is located in the posterior diencephalon abutting the third ventricle (Björklund and Skagerberg, 1979; Barraud et al., 2010; Koblinger et al., 2014; Sharples et al., 2014). These neurons are non-canonical dopaminergic cells which lack the dopamine reuptake transporter in mouse (DAT) (Koblinger et al., 2014; Yip et al., 2017). The A11 cell group appears to be evolutionarily conserved from zebrafish (Ryu et al., 2007; Lambert et al., 2012) to mammals (Barraud et al., 2010). The broader connectome of the A11 has been under investigation; however, it has been shown to receive inputs from the parabrachial nucleus, infralimbic cortex, and the bed nucleus of the stria terminalis (Abrahamson and Moore, 2001; Qu et al., 2006). One of the primary efferent targets of the A11 is the spinal cord – sending collaterals at regular intervals into the gray matter, and has been proposed to be the primary source of spinal dopamine (Björklund and Skagerberg, 1979; Commissiong et al., 1979; Skagerberg and Lindvall, 1985). While the functions of the A11 are not fully understood, it appears to have both anti-nociceptive and pro-nociceptive properties (Fleetwood-Walker et al., 1988; Megat et al., 2018), pathologically it has been associated with Restless Legs Syndrome (RLS) (Clemens et al., 2006), and 6-OHDA lesions of the area are associated with an increase in motor function (Qu et al., 2007) possibly by actions on dopamine receptor type 3 (D_3_) receptors (Clemens and Hochman, 2004; Qu et al., 2007).

One way in which A11 projections could control motor circuits is through direct modulation of spinal circuits, and indeed a sizable literature suggests that dopamine modulates the activity of motoneurons and interneurons within the lumbar spinal cord (Barrière et al., 2004; Madriaga et al., 2004; Han et al., 2007; Humphreys and Whelan, 2011; Gozal et al., 2014; Sharples et al., 2015; Sharples and Whelan, 2017). Specifically, dopamine can alter the frequency and pattern of locomotor activity, by increasing spiking frequency robustly, and decreasing both the first spike latency and after hyperpolarization amplitude (Han et al., 2007). The premotoneuronal excitatory drive onto motoneurons is also increased since dopamine can potentiate AMPA transmission in motoneurons by acting on D_1_ receptors (Han and Whelan, 2009), and increase mEPSC frequency in motoneurons (Han et al., 2007). Finally, recent evidence suggests that dopamine acts on the sodium pump in motoneurons to directly increase an ultra-slow hyperpolarization (Picton et al., 2017). In sum, dopamine can modulate spinal motor function through an array of modulatory mechanisms. The larger family of monoamines (Kiehn et al., 1999; Madriaga et al., 2004; Liu and Jordan, 2005) and trace amines (Gozal et al., 2014; Hochman, 2015), can also sculpt the pattern of activity evoked from spinal cord circuits. Sources of these monoamines include supraspinal projections (Schmidt and Jordan, 2000; Sławińska et al., 2014).

Given the effects of monoamines on spinal motor circuits, this led to our hypothesis that activation of the A11 region in the freely behaving adult mouse would lead to changes in motor behavior. We found that photostimulation of the A11 region elicits motor behavior, and that dopamine neurons lacking DAT are present in the region (Ugrumov, 2009). Part of this work was reported in an abstract and in a mini-symposium (Koblinger et al., 2015; Whelan, 2017).

## Materials and Methods

### Ethics Statement

All animal experiments were approved by the University of Calgary Health Sciences Animal Care Committee (Protocol: AC15-0016), following the Canadian Council for Animal Care guidelines. Animals were housed in a double-barrier facility for breeding.

### Animals

TH-IRES-Cre knock-in mice were used (a gift from Dr. Antoine Adamantidis, Universität Bern, Switzerland – source; EM: 00254;B6.129X1-Thtm1(cre)Te/Kieg; European Mouse Mutant Archive) as well as DAT-IRES-Cre knock-in mice were obtained from Jackson labs [B6.SJL-Slc6a3tm1.1(cre)Bkmn/J]. Reporter mice were purchased from Jackson labs [tdTomato mice: B6.Cg-Gt(ROSA)26Sortm14(CAG-TdTomato)Hze/J (Ai14)]. vGluT2-Cre-tdTomato (gift from Dr. Marie-Claude Perreault, Slc17a7-IRES2-CRE, Jackson labs) and vGAT-ChR2-eYFP [B6.Cg-Tg(Slc32a1-COP4^∗^H134R/EYFP)8Gfng/J, Jackson labs] mice were also used. We then crossed the different lines to obtain TH-IRES-Cre homozygous, TH-IRES-Cre tdTomato, and DAT-IRES-Cre tdTomato heterozygous mice. Pairs of homozygous TH-IRES-Cre (male), DAT-IRES-Cre (female) or Ai14 (male or female) genotypes were mated, and the resulting heterozygous TH-IRES-Cre; Ai14 and DAT-IRES-Cre; Ai14 male offspring were used in subsequent experiments. All mice were genotyped with DNA extracted from ear notches using the Kapa mouse genotyping kit (Kapa Biosystems, Wilmington, MA, United States) according to manufacturer’s instructions. TH-IRES-Cre mice were genotyped using mixed primer PCR employing TH-IRES-Cre-F (CCTGGTCTGGACACAGTGC), TH-WT-F (CAAGCACTGAGTGCCATTAGC) and TH-Com-R (AGAGGCCAGGAACACTCCTG). Amplification of wild-type genomic DNA yielded a 298 bp PCR product whereas amplification from the mutant locus yielded a 453 bp PCR product. Genotyping of the TH-IRES-tdTomato and DAT-IRES-Cre mice was performed similarly, using the primers recommended by the supplier (Jackson Labs, Bar Harbor, ME, United States). Mice were housed on a 12:12 h light:dark schedule (lights on at 07:00 – off at 19:00) with *ad libitum* access to food and water. All behavioral experiments were performed in male mice between 8 and 16 weeks old.

### Validation of TH-IRES-Cre Mice via Real Time qPCR

Total RNA was isolated from TH-IRES-Cre;CAG-LSL-tdTomato mouse microdissected brain regions (*n* = 7) A13, A11, and substantia nigra/ventral tegmental area (SN/VTA) using the RNeasy lipid tissue mini kit (Qiagen, Hilden, Germany) following manufacturer’s recommendations. Reverse transcription was performed using the Superscript VILO IV kit (Thermo Fisher, Waltham, MA, United States). For the negative control groups, all components except the reverse transcriptase were included in the reaction mixtures. The primers for real time qPCR were mGAPD-F (GTGAAGGTCGGTGTGAACG) and mGAPD-R (TCGTTGATGGCAACAATCTC); TH-F (CCCAAGGGCTTCAGAAGAGC) and TH-R (ATCCTCGATGAGACTCTGCC); Cre-F (ACGCACTGATTTCGACCAGGTTCG) and Cre-R (CATTCTCCCACCGTCAGTACGTGAG). Real time qPCR was performed with PowerUp SYBR green master mix (Thermo Fisher, Waltham, MA, United States) and mouse GAPDH was utilized as the reference gene. The running protocol extended to 45 cycles in fast mode consisting of 95°C for 1 s, 60°C for 30 s using a Quantstudio 3 instrument (Thermo Fisher, Waltham, MA, United States). Standard curve experiments using control mouse brain RNA were determined using a 5 log range of cDNA concentrations yielding efficiencies ranging from 90 to 110% (Bustin et al., 2009). Control reactions and those containing cDNA from the various brain regions were performed with 10 ng of template per reaction. PCR specificity was checked by dissociation curve analysis. Template controls yielded no detectable fluorescence. The relative mRNA abundance of TH and Cre in each sample were normalized to the abundance of GAPDH mRNA.

### Optogenetics

Under isoflurane anesthesia (1.5 %), mice were secured in a stereotaxic apparatus (Stoelting Co., Wood Dale, IL, United States), and glass capillaries were lowered into the brain of TH-IRES-Cre or TH-IRES-Cre; Ai14 mice (AP -2.3 mm; ML -0.1 mm from the bregma; DV -2.9 mm from the dura). Recombinant AAV carrying a fluorescently tagged channelrhodopsin 2, ChR2-eYFP (Addgene plasmid 20298, pAAV-EF1a-double floxed-hChR2(H134R)-eYFP-WPRE-HGHpA; titre: 3 × 10^12^ GC/ml; Virus Vector Core, UNC, Lot # AV4844B) or eYFP (Addgene plasmid 20296, pAAV-EF1a-double floxed-eYFP-WPRE-HGHpA; 4 × 10^12^ GC/ml; Virus Vector Core, UNC, Lot # AV4842C) were pressure injected using the Nanoject II apparatus (Drummond Scientific Company, Broomall, PA, United States). 210 nl of the virus were injected bilaterally, with a total volume of 420 nl per mouse.

Mice were returned to their home cage and left for at least 14 days to allow for transfection of the viral construct into A11 neurons. For optogenetic experiments, mono-fiber optic cannulas were stereotactically implanted into the A11 area (MF2.5-FLT, Doric, Quebec, QC, Canada) (AP -2.3 mm; ML, -0.1 mm from the bregma; DV -2.7 mm from the dura). Fiber optic cannulas were secured in place using dental cement (Metabond, Parkell, Brentwood, NY, United States and Dentsply, York, PA, United States). Mice were allowed to recover for at least 3 days before behavioral testing. We performed *post hoc* histology to confirm A11^ChR2^ transfection and ferrule placement.

### Whole Cell Patch Experiments

In a separate series of experiments, we measured inward currents and spike activity using *in vitro* slice preparations to examine the effectiveness of A11^ChR2^ transfection. Young male TH-IRES-Cre; Ai14 mice (4–6 weeks postnatal) were deeply anesthetized with isoflurane and decapitated. Brains were then rapidly removed and immersed in 4°C slicing solution containing, in mM: 87 NaCl, 2.5 KCl, 0.5 CaCl_2_, 7 MgCl_2_, 25 NaHCO_3_, 25 D-glucose, 1.25 NaH_2_PO_4_, 75 sucrose saturated with 95% O_2_/5% CO_2_. 250 μm coronal sections were obtained using a vibratome (Leica), and allowed to recover for 1 + h in 95% O_2_/5% CO_2_ saturated, 30°C artificial cerebrospinal fluid (aCSF) containing (in mM): 126 NaCl, 2.5 KCl, 26 NaHCO_3_, 2.5 CaCl_2_, 1.5 MgCl_2_, 1.25 NaH_2_PO_4_, 10 glucose. All recordings took place in aCSF at 30–32°C perfused at a rate of 1 mL/min, with DNQX (10 μM, Tocris) or picrotoxin (100 μM, Sigma) applied via perfusion pump. Neurons were visualized with an upright microscope fitted with differential interference contrast and epifluorescence optics (UVICO, Rapp OptoElectronic) and camera (AxioCam MRm). Borosilicate pipettes (3–5 MΩ) were filled with internal solution containing (in mM) 108 K-gluconate, 2 MgCl_2_, 8 Na-gluconate, 8 KCl, 1 K_2_-EGTA, 4 K_2_-ATP, 0.3 Na_3_-GTP, 10 mM HEPES, 0.2 Alexa-488 hydrazide and 10 mg/mL biocytin. A fiber optic cable (105 μm core diameter) was placed 1–2 mm from the A11 using a manipulator to deliver light from a laser (473 nm, OptoGeni 473, IkeCool Corporation). Light intensity was measured by a Photodiode Power Sensor (Thorlabs). Maximally, 2.5 mW light was delivered to the tissue. Signals were amplified (Multiclamp 700B, Molecular Devices), low pass filtered at 1 kHz, digitized at 10 kHz (Digidata 1322, Molecular Devices, San Jose, CA, United States), and recorded (pClamp 9.2, Molecular Devices) for offline analysis of evoked or spontaneous synaptic currents (Clampfit, Molecular Devices; MiniAnalysis, Synaptosoft).

### Behavior

For *in vivo* experiments the light source (473 nm, LRS-0473-GFM, Laserglow Technologies, Toronto, ON, Canada) was connected to an implanted ferrule with a fiber optic cable (200 μm core diameter, Doric Lenses, Quebec City, QC, Canada). The laser was controlled by TTL pulses delivered using a Master 8 pulse stimulator (A.M.P.I., Jerusalem, Israel). The blue light was delivered for 3 min (20 Hz, 10 ms pulse width, 15 mW).

Each mouse was tested for 9 min (3 min pre, 3 min light, 3 min post) for five consecutive days. Behavior was recorded using a vertically mounted video camera and *post hoc* analyzed with the TopScan video tracking software (Clever Sys Inc., Reston, VA, United States). For c-Fos immunolabelling, animals were sacrificed 2 h after light stimulation. Behavioral testing was performed in a 70 cm × 70 cm × 50 cm high open field chamber with opaque walls. Each mouse was habituated to the chamber for 3 days for 1 h per day before testing. On test days, mice were habituated for 30 min before testing and trials were performed at the same time of day. Mice were transferred to and from a behavioral testing room for day-to-day testing. Mice were housed singly in standard cages (SafeSeal Plus Mouse Green Line GM500, Techniplast, Italy, 501 cm^2^ floor) with no environmental enrichment.

The Clever Sys tracking software divided movement into three categories:

(1) Immobility/No movement: Immobility is defined by both speed and motion measures (animal body change in consecutive frames expressed as a percentage). Immobility bouts over 15 successive frames were estimated when speed was <60 mm/s and the change in motion was <1% change in animal body position.
(2) In Place Activity: Animal stays in one place with some observed activity such as grooming, sniffing, stretching, rearing, but the animal is not immobile.
(3) Locomotion. Distance traveled: The mouse physically moves or displaces itself from one location to another with a minimum prescribed speed of greater than 60 mm/s and a minimum distance of 100 mm. Locomotor bouts: the number of times animal moves from one place to another place. If the animal continuously moves, it is counted as one single bout. Locomotor duration: the amount of time the mouse spends in locomotion.

### Immunohistochemistry

Mice were sacrificed with isoflurane (2%) and transcardially perfused with phosphate-buffered saline (PBS), followed by 10% formalin in PBS. Brains were placed in 10% formalin for 12 h followed by 30% sucrose phosphate buffer (PB) for cryoprotection. 30 μM coronal brain sections were obtained using a cryostat (Leica CM1850 UV, Leica Biosystems, Richmond Hill, ON, Canada). The sliced brain sections were collected in a staggered fashion and placed into four consecutive wells. Rinses were performed before and between incubations with 0.1 M PBS, followed by one 20 min wash in Tris-buffered saline containing Triton (TBSt; pH 7.4, with 0.1% TritonX-100). Sections were incubated in blocking solution (5% donkey and 5% goat serum in PBS) for 1 h, and blocking solution was used in subsequent antibody incubations. The primary antibodies used were rabbit anti-TH, sheep anti-TH (1:1000, Abcam Inc., Toronto, ON, Canada), chicken anti-GFP (1:1000, Aves Laboratories, Tigard, OR, United States), rabbit anti-c-Fos (1:1000, EMD Millipore, Billerica, MA, United States), rabbit anti-RFP (1:5000, Rockland Immunochemicals Inc, Limerick, PA, United States) and rabbit anti-AADC (1:250, Novus Biologicals, Littleton, CO, United States). The secondary antibodies used were Alexa-564-conjugated donkey anti-rabbit, Alexa-488-conjugated donkey anti-sheep, Alexa-488-conjugated goat anti-chicken, Alexa-647-conjugated donkey anti-sheep (1:1000, Molecular Probes, Burlington, ON, Canada), biotinylated donkey anti-rabbit and Cy3-conjugated streptavidin (1:500, Jackson Immuno Research, West Grove, PA, United States). Free-floating brain sections were then mounted onto Superfrost^TM^ slides, coated with Vectashield^TM^ (H-1000, Vector, Burlingame, CA, United States) and cover-slipped. Fluorescent images were collected using the following microscopes; Nikon Eclipse C1si spectral confocal microscope, Nikon A1R MP+ and Olympus BX51. The objectives used were 4X (NA 0.13), 20X PLAN APO DIC (NA 0.75), 20X PLAN FLUOR (NA 0.75), 60X PLAN APO IR (NA 1.27) for Nikon Eclipse C1si spectral confocal microscope. The lasers used were centered on 488 nm (515/30 nm emission filter) and 561 (590/50 nm emission filter). The objectives used were 10X DIC L N1 (NA 0.30), 20X PLAN FLUOR MImm DIC N2 (NA 0.75), 60X PLAN APO IR WI DIC N2 (1.27) for the Nikon A1R MP+ microscope. The lasers used were centered at 403 nm, (450 nm emission filter) 488 nm (525 nm emission filter), and 562 nm (595 nm emission filter), respectively using pinhole radius of 12.7–21.5 microns on Nikon A1R MP+ microscope. The 20X images were taken with z-step 0.5 or 1 μm, 60X with z-step 0.15 μm. Stacked images were acquired by averaging four frames with a resolution of 1024 × 1024 or 512 × 512. Some images were acquired using a Panoramic FLASH II digital slide scanner (3DHISTECH inc., provided by Quorum Technologies) equipped with a Lumencor SPECTRA light source and PCO.edge sCMOS camera. Off-line image processing included maximal intensity projections conducted using NIS-Elements Advanced Research Version 4.10 as well as adjustments of brightness and contrast in Adobe Photoshop. Cell counting was accomplished by a person blinded to the study using ImageJ (NIH Image, Bethesda, MD, United States). We counted five sections containing the area of interest per animal.

### Data Analysis and Statistics

Statistical analyses were performed in GraphPad Prism 6. Unpaired Student’s *t*-tests were conducted comparing between two independent groups (e.g., A11^eYFP^ vs. A11^ChR2^). When assumptions of normality were violated, a Mann–Whitney test was performed instead of a *t*-test. A two-way repeated measures analysis of variance (ANOVA) was conducted to examine changes between two groups (i.e., A11^eYFP^ vs. A11^ChR2^) across time. When a significant main effect or interaction was found, Tukey *post hoc* comparisons were conducted. When assumptions of normality were violated on the ANOVA, non-parametric Friedman tests were done with a Dunn’s multiple comparison tests when significant main effects were found. Repeated measures one-way ANOVA (RM-ANOVA) tests were conducted to examine changes within a group (i.e., A11^eYFP^ vs. A11^ChR2^) in different conditions (before, during, just after light-activation of the A11). When a significant main effect or interaction was found, Bonferroni multiple *post hoc* comparisons tests were conducted. Data are reported as the mean ± standard deviation (SD) and *P* < 0.05 values were considered significant. When data did not pass normality tests, the median is reported with 25–75% ranges. When histograms were used (**Figure 6**) both the median and mode were used to report data.

## Results

### Classes of Cells Within the A11 Region

**FIGURE 1:**
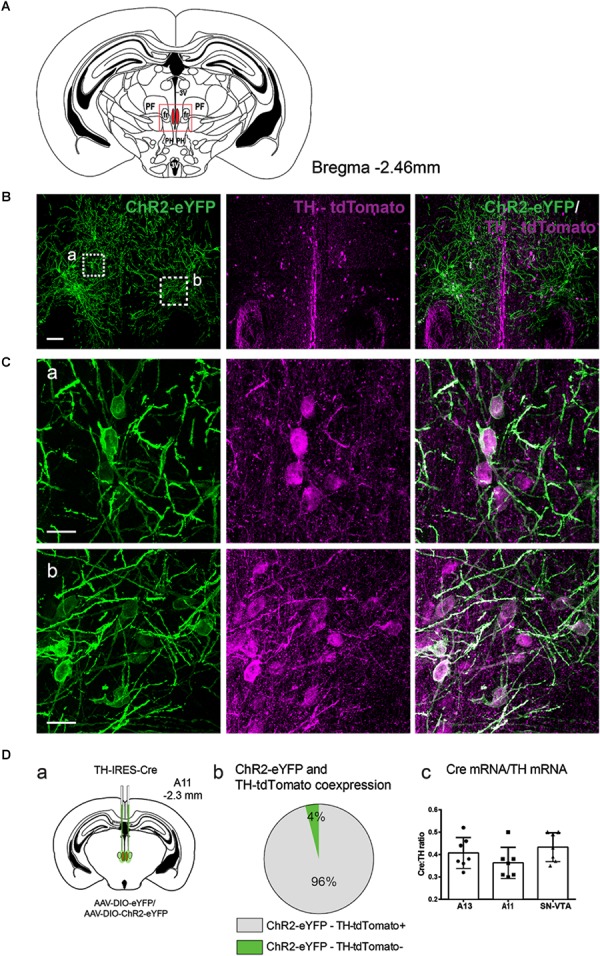
Anatomical characterization of TH-IRES-Cre A11 neurons following transfection with AAV-DIO-ChR2-eYFP. **(A)** Diagram showing the middle region of the A11 area in the mouse (Bregma –2.46). The red frame represents the area where representative micrographs were taken. 3V, third ventricle; PF, parafascicular thalamic nucleus; fr, fasciculus retroflexus; PH, posterior hypothalamic nucleus. **(B)** TH-IRES-Cre tdTomato mice expressing channelrhodopsin-2 – enhanced yellow fluorescent protein (ChR2-eYFP) in A11. Confocal image showing A11 neurons expressing ChR2-eYFP bilaterally (stitched image consisting of 4 20X images). Scale bar 100 μm. **(C)** Higher magnification (60X zoom) of boxed areas **(a,b)** in **B**, Scale bar 20 μm. **(Da)** Schematic map showing the injection site of virus with the Cre-dependent ChR2 construct tagged with ChR2-eYFP or eYFP alone into the A11 of TH-IRES-Cre mice. **(Db)** Pie graph showing that 96% of ChR2-eYFP positive cells in A11 co-express tdTomato (*n* = 4). **(Dc)** Quantitative real-time PCR analysis of ratio of Cre mRNA to that of TH mRNA in A13, A11 and substantia nigra/ventral tegmental area (SN/VTA) brain regions.

**FIGURE 2:**
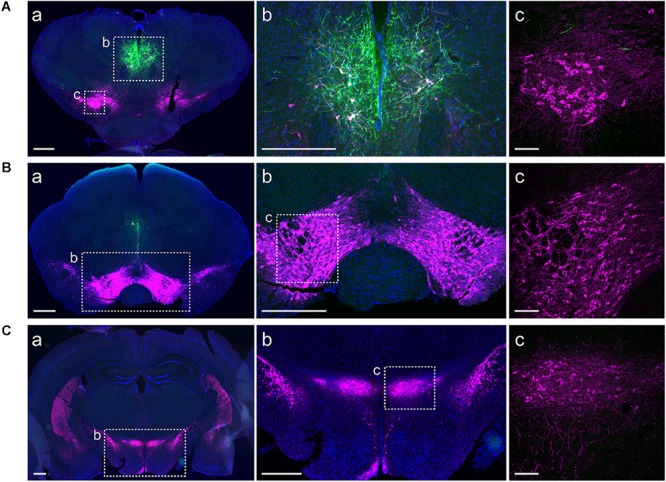
ChR2-eYFP expression is restricted to A11 area. Immunohistochemistry targeted against eYFP (green) and TH (magenta) showing restricted ChR2-eYFP expression to A11. **(Aa,b)** ChR2-eYFP expression in A11 following Cre-dependent viral expression (DAPI, blue was used to label the cell nuclei). **(Ac)** Higher magnification (20X) of boxed TH^+^-IR neurons in substantia nigra pars compacta. **(Ba,b,c)** No ChR2-eYFP expression in the ventral tegmental area (VTA). **(Ca,b,c)** No ChR2-eYFP expression in A13. Images **(Aa,b, Ba,b, Ca,b)** acquired with Olympus slide scanner 20X magnification, scale bar 500 μm; **(Ac, Bc, Cc)** Higher magnification confocal images of boxed area in **b (Ab, Bb, Cb)**, scale bar 100 μm.

We used a viral approach to identify classes of cells within the A11. The viral transfection efficiency was confirmed by injecting an AAV-DIO-ChR2-eYFP construct into A11 region in TH-IRES-Cre-tdTomato reporter mice (**Figures 1A–C**, *n* = 4). We found that 96% (SD = 1.7%) of ChR2-eYFP neurons expressed TH-tdTomato (**Figure 1D**) in the A11 region. To confirm that Cre expression levels correlated with the expression levels of endogenous TH, real time qPCR analyses were performed on RNA isolated from TH-expressing brain regions A13 (A), A11 (B), and SN/VTA (C) (**Figure 1D**). Expression of TH and Cre, respectively, were normalized to the expression of the reference gene GAPDH. The normalized expression of Cre was consistently lower than that of TH, with levels ranging from 36 to 43%, depending on the brain region. All of the animals analyzed were heterozygous for the Cre transgene so the expected result was that Cre mRNA levels would be 50% of TH mRNA levels. Based on these results we expect that Cre mRNA levels will be close to TH mRNA levels in homozygous TH-IRES-Cre mice. Next, we confirmed in TH-IRES-Cre mice that the viral injections were restricted to the A11 region. **Figure 2** shows that the expression of ChR2-eYFP^+^ was spatially restricted to neurons from the A11 region (**Figure 2A**) following Cre-dependent viral expression. Other neighboring TH expressing regions, like the VTA, substantia nigra pars compacta and A13 did not show evidence of eYFP reporter expression (**Figures 2Ac,Ba–c,Ca–c**, respectively).

**FIGURE 3:**
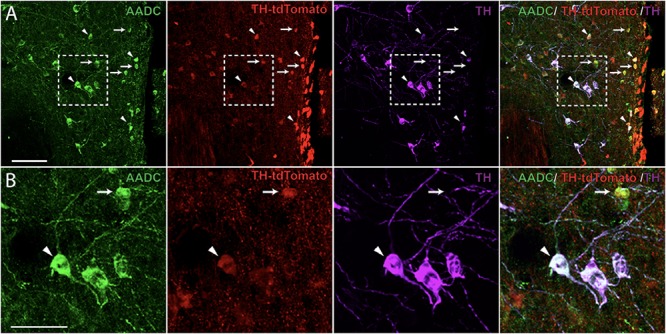
Aromatic L-amino acid decarboxylase (AADC) expression in A11 TH-IR neurons. **(A)** Immunohistochemistry targeted against AADC (green) and TH^+^-IR (magenta) as well as native TH-tdtomato (red) expression showing that a proportion of A11 TH-tdTomato^+^/TH^-^-IR neurons were also AADC^+^ in TH-IRES-Cre tdTomato mice. 20X magnification, scale bar 100 μm. **(B)** Enlarged image showing AADC^+^/TH-tdTomato^+^/TH^-^-IR (arrows) and AADC^+^/TH-tdTomato^+^/TH^+^-IR neurons (arrowheads). 20X magnification, scale bar 50 μm.

Next, we examined the co-localization of TH^+^-IR cells with A11^ChR2^ cells to identify the neurotransmitter phenotype of the cells transfected within the A11 region of TH-IRES-Cre mice. Similar to recent reports (Lammel et al., 2015) we found that 54% of A11^ChR2^ expressing cells were co-localized with TH^+^-IR (*n* = 4, ChR2 mean = 132, SD = 32.01, TH^+^-IR mean = 105.5, SD = 11.03; co-localizing mean = 71.75, SD = 15.97, data not shown). In our previous work, we established that the large diameter cells which constitute the A11 region in mice are a non-canonical class of dopaminergic neurons that lack the Dopamine Transporter (DAT) (Koblinger et al., 2014) which was replicated in a report that used a DAT-GFP reporter line (Yip et al., 2017). We further confirmed these findings using our DAT-Cre-tdTomato reporter line (data not shown). We then tested for non-classical types of monoaminergic cells within the A11 region. These neurons only express the AADC enzyme and can produce various monoamines and trace amines (Ugrumov, 2009). We found evidence for AADC cells lacking TH in the A11 region (**Figure 3**) which suggest the presence of putative D-cells in the A11 region (Jaeger et al., 1983). In TH-tdTomato^+^ mice, we found that 35% of TH-tdTomato^+^/TH^-^-IR neurons were also AADC^+^ in the A11 region (*n* = 5, TH-tdTomato^+^/TH^-^-IR mean = 185.8, SD = 129.1, TH-tdTomato^+^/TH^-^-IR/AADC^+^ mean = 64.4, SD = 38.14; **Figure 3**). Taken together these results suggest that approximately 70% of transfected cells are capable of releasing dopamine and other monoamines such as serotonin (5-HT) or trace amines.

**FIGURE 4:**
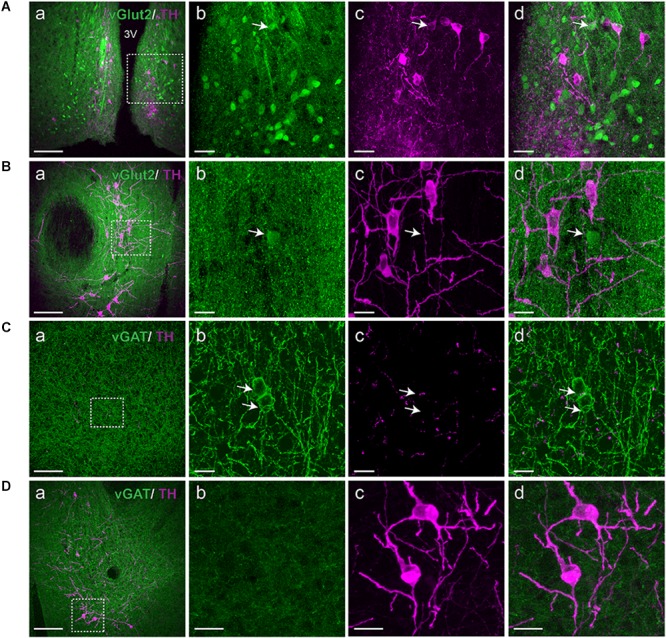
A11 TH^+^-IR neurons lack vGluT2 and vGAT expression. **(A,B)** Immunohistochemistry targeted against TH (magenta) in a vGluT2-reporter (green) mouse. (Aa) vGluT2 expressing neurons are plentiful in the arcuate nucleus of hypothalamus, scale bar 200 μm. **(Ab–d)** Higher magnification of boxed area **(Aa)**, Scale bar 20 μm. vGluT2 is present in the arcuate nucleus of hypothalamus which served as a positive control. Arrow indicates a double positive TH^+^-IR A11 neuron expressing vGluT2. **(Ba)** TH^+^-IR neurons in A11 region, scale bar 200 μm. **(Bb–d)** Higher magnification of boxed area **(Ba)**, scale bar 10 μm. We observed a few vGluT2 expressing neurons in the A11 region but these neurons lacked TH^+^-IR (arrow). **(C,D)** Immunohistochemistry targeted against TH (magenta) in a vGAT-reporter (green) mouse. **(Ca)** Large number of vGAT expressing GABAergic interneurons are observed in cortex, scale bar 200 μm. **(Cb–d)** Higher magnification of boxed area **(Ca)**, scale bar 10 μm. Arrows indicate GABAergic interneurons expressing vGAT and TH^+^-IR fibers and puncta are also observed. **(Da)** TH^+^-IR neurons in A11 region, scale bar 200 μm. **(Db–d)** Higher magnification of boxed area **(Da)**, scale bar 10 μm. TH^+^-IR neurons in A11 region lack any vGAT expression.

Since there is evidence of localization of dopamine with co-transmitters such as peptides, glutamate or GABA, we asked whether co-localization of dopamine neurons in the A11 with GABA or glutamate could be detected. **Figure 4** illustrates there is evidence of a small number of vGluT2^+^ cells within the A11 region. However, there was no vGluT2^+^-TH^+^-IR co-localization in the A11 region, compared to the arcuate nucleus of hypothalamus (**Figures 4A,B**). While vGluT2 was the most likely target subpopulation we also examined Allen Brain Atlas data and found strong expression of vGluT3, however, no evidence for vGluT1 staining in the A11 vicinity was found. Our data indicates a lack of vGAT colocalization in TH^+^-IR neurons of A11 (**Figures 4C,D**). We did see evidence for some vGAT^+^ somata within the A11 region.

### Photostimulation of the A11 Region Increases Motor Activity

Given that the viral injections were restricted to the A11 region, we tested the feasibility of using TH-IRES-Cre mice as a tool to photostimulate A11 neurons.

**FIGURE 5:**
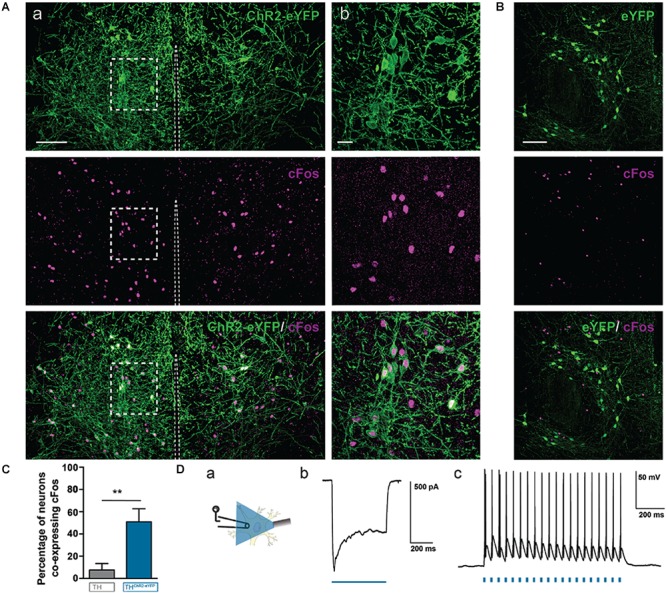
Photostimulation of A11 cells induces c-Fos protein expression in A11 ChR2-eYFP neurons *in vivo* and action potentials *in vitro*. ChR2-eYFP expressing (*n* = 4) mice and eYFP control (*n* = 4) mice were stimulated with 473 nm light at 15 mW with 20 Hz, 10 ms pulses, 2 h prior to sacrifice. **(Aa)** A11 neurons from ChR2-eYFP light stimulated mouse (stitched image consisting of 4 20X images; scale bar 100 μm). **(Ab)** Higher magnification (60X zoom) of boxed area (scale bar 20 μm). **(B)** Photostimulation of A11 eYFP neurons (no ChR2) *in vivo* does not result in significant co-localization of c-Fos in A11-eYFP^+^ cells (scale bar 100 μm). **(C)** Bar graph summarizing changes in the percentage of neurons co-expressing c-Fos in ChR2-eYFP (*n* = 4) versus eYFP (*n* = 4). **(D)** Real-time optical stimulation with 473 nm light reliably elicits spikes in A11 neurons *in vitro*. Example of a photocurrent recorded in voltage-clamp mode, elicited by 473 nm blue light **(Db)**. Trains of light pulses (10 ms pulse-width) at 20 Hz frequency evoked spike activity **(Dc)**. **(Da)** Schematic showing approach. ^∗∗^*P* < 0.01.

First, we tested, using brain slices, if the A11^ChR2^-cells could elicit sodium (Na^+^) spikes or currents following photostimulation. Pulses of blue light (473 nm) were delivered via an optical fiber, which successfully elicited photocurrents in A11^ChR2^ cells (**Figure 5**). We also determined that trains of Na^+^ spikes were reliably elicited at frequencies up to 20 Hz (**Figure 5Dc**), which was the frequency we adopted for *in vivo* studies.

We then asked whether light pulses directed to the A11 region *in vivo* could also successfully activate A11^ChR2^-cells. We implanted a fiber optic cannula unilaterally 0.1 mm lateral to the midline just above A11 region to bilaterally stimulate A11. We photostimulated both A11^ChR2^ or A11^eYFP^ mice at 20 Hz for 3 min (same parameters as for behavior), sacrificed them 2 h later to allow for c-Fos expression and prepared brain slices to probe for c-Fos^+^-expression, which is increased in neurons following depolarization (**Figures 5A,B**). We found that in 10 animals (ChR2 *n* = 5, eYFP *n* = 5) A11^ChR2^-cells were more likely to be also c-Fos^+^ compared to cells infected with control eYFP ^+^ construct (**Figure 5C**, ChR2 *n* = 5, eYFP *n* = 5, *U* = 0, *P* = 0.004, Mann–Whitney test). These data suggest that the A11^ChR2^-cells can be photo-activated *in vitro* and *in vivo* and that the neurons from the A11 region that are infected with control eYFP^+^ construct are not activated by the same light stimulation parameters.

**FIGURE 6:**
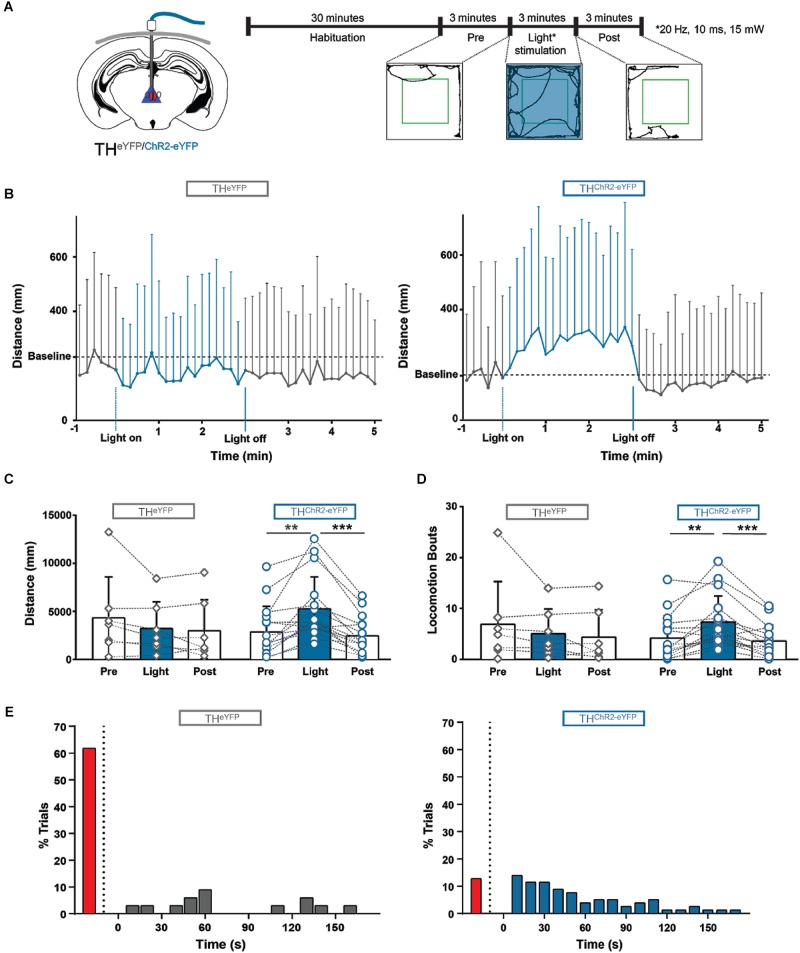
Photostimulation of A11 in awake behaving mice initiates and modulates locomotion. **(A)** Schematic showing the implantation site of the light ferrule, along with a schematic of the experimental plan. **(B)** Locomotor distance was significantly increased during photostimulation in ChR2 animals compared to eYFP-control mice (averaged over 5 days). Baseline was corrected. **(C,D)** ChR2 mice displayed significantly more distance traveled **(C)** and locomotion bouts **(D)** during blue light stimulation compared with light-off conditions. There was no significant difference between the three conditions in eYFP control mice (*n* = 7) (average over 5 days). **(E)** 87.3% of ChR2 trials (blue bars) crossed the threshold during photostimulation while only 38.2% of the eYFP trials (gray bars) crossed the threshold (5 days, all trials), looking at individual trials the median time to cross the threshold was 40 s for ChR2 mice, and 60 s for eYFP mice. The red bars on the left of both graphs indicate the percentage of trials where the threshold was not crossed. These data suggest that photostimulation of A11 can initiate and modulate locomotion. ^∗∗^*P* < 0.01; ^∗∗∗^*P* < 0.001.

Next, we addressed the functional role of A11 neuronal activation in freely moving mice. We assessed movement in the open field test in animals expressing A11^ChR2^ or in A11^eYFP^. The mice were habituated to the open field for 1 h daily for 3 days and then tested for a fixed period on each of five consecutive days. Prior to testing, mice were habituated to the open field for 30 min with optical fibers attached. We recorded baseline activity 3 min prior to light activation, directly followed by 3 min of 20 Hz, 10 ms pulse width of light (473 nm) and 3 min of post-light activation (**Figure 6A**).

In the A11^ChR2^ cohort only, qualitatively we noticed that animals reacted immediately to the light by changing behavior in a variety of ways (e.g., postural changes, starting to groom, turning, sniffing, stretching, and rearing but the animal is neither locomoting nor immobile). Collectively, these behaviors were quantified as changes in “in-place activity.” When we compared the effect of light between the groups (A11^ChR2^ and A11^eYFP^, baseline corrected; difference = value - baseline) we found significant differences between the groups for in place activity (A11^ChR2^ *n* = 16, 803 ± 741 mm; A11^eYFP^ -391 ± 503 mm; *n* = 7, *U* = 11, *P* = 0.0007, Mann–Whitney test; data not shown).

These animals would then generally begin to locomote after engaging in these activities (see **Supplementary Video 1**). When we further examined these changes in the A11^ChR2^ cohort, photostimulation increased the distance traveled by 86.0% (**Figure 6C**; *n* = 16, *Q* = 18.9, *P* < 0.0001, Friedman test) and the total number of locomotor bouts by 75.6% (**Figure 6D**; *n* = 16, *Q* = 16.6, *P* = 0.0002, Friedman test, see methods for definitions). In contrast, this was not observed in the A11^eYFP^ control group (**Figure 6C**, *n* = 7, Q = 2.0, *P* = 0.5; **Figure 6D**, *n* = 7, *Q* = 1.5, *P* = 0.5, Friedman test). When we compared the effect of light between the groups (A11^ChR2^ and A11^eYFP^, baseline corrected, difference = value - baseline) we found significant differences for the following parameters: locomotion (A11^ChR2^ *n* = 16, 1551 ± 1503 mm; A11^eYFP^ -149 ± 665 mm; *n* = 7, *U* = 10, *P* = 0.0005, Mann–Whitney test), and bouts of locomotion (A11^ChR2^, *n* = 16, 1.88 ± 4.09 bouts; A11^eYFP^, -3.18 ± 3.36 bouts; *n* = 7, *U* = 13, *P* = 0.001, Mann–Whitney test). We did not observe an effect of day [2-way ANOVA with repeated measures, *F*(4.6) = 0.04, *P* = 1].

Next, we determined the latency for changes in movement behavior after photostimulation. For this, we averaged the data (distance in mm) into 10 s bins and set a threshold (mean of the pre-light distance traveled over 3 min, + two times SD) for each individual trial and determined the latency of crossing the threshold. We examined all trials (*n* = 80, all days, all animals) and found that 87.3% of the A11^ChR2^ trials crossed threshold during light activation with a median latency of 40 s (25–75% percentile 20–80, **Figure 6E**). In contrast, only 38.2% of the A11^eYFP^ mice trials (*n* = 35, all days, all animals) crossed threshold with a median latency of 60 s (25–75% percentile 48–130 **Figure 6E**).

When we examined all photostimulation trials on a trial-to-trial basis, there was no significant correlation between basal locomotor activity and the light mediated effect (distance traveled (mm); *r* = -0.1, *P* = 0.1) due to variability in the data (mean = 43.80 mm, SD = 272.2, median = 2.6 mm). Taken together our data suggest that photostimulation of A11 affects both the movement during locomotion as well as the in-place activity.

## Discussion

Previous work has shown that the diencephalic A11 cell group contains neurons that project to the spinal cord and its effects are associated with pain control (Fleetwood-Walker et al., 1988), migraine (Charbit et al., 2009), cataplexy (Okura et al., 2004), and possibly a role in RLS (Clemens et al., 2006). Our work provides evidence that activation of A11 increases motor behavior, including locomotor and non-locomotor events. The A11 was shown to depress firing of dorsal horn neurons responding to noxious input (Fleetwood-Walker et al., 1988; Taniguchi et al., 2011), through a D_2_ receptor-mediated mechanism (Tamae et al., 2005). Similar effects on trigeminal nociception have been also reported (Abdallah et al., 2015). But dopamine has also been shown to be pro-nociceptive through D_5_ receptors expressed in the spinal cord (Megat et al., 2018). This complex sensorimotor role is shared with other monoamines such as 5-HT and noradrenaline (Schmidt and Jordan, 2000; Millan, 2002), and is partly dependent on the diversity of monoamine receptor subtypes. Indeed, this sensorimotor modulation may be related to expression of movement as hypothesized for RLS (Clemens et al., 2006). As outlined below, dopamine’s actions on motor circuits are also diverse. This points to a role in both pain and motor function where the actions of dopamine can modulate multiple circuits within the spinal cord to fine-tune sensorimotor function. A further diversity of A11 actions could be linked to the sex of the animals. Our work used male mice and it is possible that results in females may differ, since work in rats has shown a sexual dimorphism in the A11 descending projections (Pappas et al., 2008, 2010) and recent work show spinal D_5_ receptors in the dorsal horn contributing to hyperalgesia in male but not female mice (Megat et al., 2018). Since male rats have a greater density of A11 fibers in the ventral horn it is possible that A11 stimulation in males would show greater motor effects compared to females.

### Mode of Action

Our previous work established that in mice, the A11 is primarily dopaminergic but lacks DAT (Koblinger et al., 2014) which has also been confirmed using DAT reporter mice (Yip et al., 2017). In the A11 system, this DAT^-^ mechanism may increase synaptic dopamine concentration, augmenting dopamine’s actions within the spinal cord.

Data from the neonatal rat and mouse shows that bath application of dopamine restricted to the thoracolumbar spinal region modulates fictive locomotor patterns (Kiehn and Kjaerulff, 1996; Jiang et al., 1999; Whelan et al., 2000; Barrière et al., 2004; Christie and Whelan, 2005; Gordon and Whelan, 2006; Humphreys and Whelan, 2012; Sharples et al., 2015; Sharples and Whelan, 2017). This dopamine-evoked excitation of mammalian spinal locomotor circuits occurs through D_1/5_ receptors (Maitra et al., 1993; Seth et al., 1993; Barrière et al., 2004; Madriaga et al., 2004; Gordon and Whelan, 2006; Han et al., 2007) although a role for D_2/3/4_ receptors exists (Humphreys and Whelan, 2012). In particular, D_1/5_ receptors appear to enhance the stability of ongoing locomotion but D_2/3/4_ receptors appear to contribute to a slowing of the rhythm (Sharples et al., 2015), and may access part of the pattern generator through recurrent collaterals (Humphreys and Whelan, 2012). In adult spinalized mice, D_1_ receptors appear to contribute more than D_5_ receptors to locomotion, based on the use of D_5_ receptors KO mice (Lapointe et al., 2009). Since this study used a mixed cohort of male and female mice, and recent evidence suggests that D_5_ receptors are present in higher numbers in the spinal cord of male mice (Megat et al., 2018), a role for D_5_ receptors in locomotion cannot be excluded. Dopamine fibers projecting from the diencephalon (A11 region) are present in the ventral horn of the mouse adult spinal cord, an area where dopamine receptors and motor circuits are located (Yoshida and Tanaka, 1988; Weil-Fugazza and Godefroy, 1993; Qu et al., 2006; Zhu et al., 2007). The release of dopamine and its metabolites occurs in the ventral horn of neonatal rats during fictive locomotion (Jordan and Schmidt, 2002) and in the spinal cord of adult rats following walking (Gerin and Privat, 1998). Moreover, administration of D_1_ agonists in adult mice with a complete thoracic injury elicited bouts of stepping (Lapointe et al., 2009). Finally, the critical role played by dopamine transmission in locomotion is highlighted by the fact that L-DOPA-elicited air stepping in intact neonatal rats is blocked by intrathecally introduced dopamine receptor antagonists (Sickles et al., 1992; McCrea et al., 1997). Work by several groups (Fleetwood-Walker et al., 1988; Taniguchi et al., 2011) has shown that stimulation of A11 in adult animals has effects on nociception, acting both pre- and post-synaptically on neurons within the dorsal horn of the spinal cord (Taniguchi et al., 2011), and inhibited by dopaminergic antagonists. Taken together, these data suggest that A11 activation can influence spinal cord circuits via dopamine, and dopamine can have several actions on spinal motor circuits.

Like many nuclei the A11 is known to project to other areas of the brain, including the amygdala, prefrontal cortex (Takada et al., 1988; Takada, 1990, 1993), and dorsal raphe (Peyron et al., 1995). Indeed diencephalon-specific pathways to the brainstem, including the medullary reticular formation and the mesencephalic locomotor region have been shown in the past (Sinnamon and Stopford, 1987; Sinnamon et al., 1987). We expect therefore that the A11 connectome is more extensive than has been published. Therefore while spinal cord connections may be responsible for the observed effects, A11 collaterals projecting to other brain regions may also be involved. Within the A11 we found neurons that were positive for vGluT2 and negative for vGAT. But we did not find cells that co-localized dopamine and vGluT2. However, vGluT3 neurons are found in the A11 vicinity (Allen Brain Atlas) so it is possible that dopamine neurons could still contain glutamate. Our data show some vGAT^+^ positive neurons in the A11 region, agreeing with data from the Allen Brain Atlas, and we saw no vGAT^+^-TH^+^-IR neurons.

### Effects on Motor Activity

We found that photostimulation of the A11 region produced immediate responses that, although varied, always led to locomotion. The delay in the initiation of locomotion is consistent with early reports of stimulation of this region (Mori et al., 1989; Sławińska and Kasicki, 1995). There are several possibilities for this delay. The A11 network may form a reverberating positive-feedback local network resulting in an increase in spike output over time, and the fact that vGluT2 neurons are in the A11 region point to this being a possibility. We do not feel that the delay may be due to fast inhibitory neurons being recruited since our data do not show vGAT^+^ somata within the A11. On the other hand, low concentrations of dopamine bind to high-affinity D_2/3/4_ receptors, producing inhibition. At higher dopamine concentrations, expected with prolonged spiking within the A11, a switch to excitation could occur (Clemens et al., 2012). Alternatively, an interesting observation is that within the isolated spinal cord preparation of the rodent, bath application of dopamine produces a delayed activation of the network often being an order of magnitude slower compared to 5-HT application (Gozal et al., 2014). An action of dopamine on trace amine receptors (TAAR1) has been proposed to account for this delay in the rat (Bunzow et al., 2001; Gozal et al., 2014) and a similar mechanism operating *in vivo* could partly account for the delays noted here. A component of the A11 cells activated were presumably D-cells (Jaeger et al., 1983), which lack TH but contain AADC, based on the Allen Brain Atlas, and our own work. Little is known about the storage and release of dopamine (or other transmitters) from these cells. Work by Weihe et al. (2006) shows evidence of hypothalamic AADC^+^/TH^-^ cells that lack VMAT2. Therefore neurotransmitter release from D-cells likely occurs differently from the standard vesicular-based dopamine release. Recent evidence shows that the hypothalamic neurons containing glutamate, GABA, and dopamine cells can be subdivided into 62 distinct subpopulations where neuropeptides are co-localized in a distinct manner (Romanov et al., 2017). While our data show that the A11 contains a small number of vGluT2 neurons we did not see any co-expression with TH^+^ neurons, but vGluT3 expression was not tested here, and is observed in the A11 region, therefore glutamate co-localization in TH^+^ neurons may still be possible. Our RT-PCR data showed that the levels of Cre mRNA are between 36 and 41% of TH mRNA in heterozygous TH-tdTomato mice, which is close to our expectations of 50% levels. Slightly lower levels of Cre mRNA may be due to the possibility that the locus of TH transcription is more active than the locus of Cre transcription. On the other hand, this supports the idea of active transcription of TH mRNA in the absence of the translation of TH protein which was highlighted in a recent report (Meng et al., 2018). In this regard, we feel that future attempts using other techniques such as double fluorescent in situ hybridization or single cell RNA-seq may provide additional insights on molecular and cellular diversity in phenotypes of TH cells in the A11. The most conservative interpretation of our data is that at a minimum 54% of ChR2-TH^+^ neurons were dopaminergic, and a possibility remains that this percentage could be higher. Since we did not observe co-localization of vGAT or vGluT2 with TH^+^-IR, we conclude that the stimulated population includes mainly dopaminergic neurons, D-cells, and an undefined neuronal population.

## Conclusion

This is the first study to show that stimulation of the A11 has pro-locomotory effects. This adds to the hypothesized role of the A11 in RLS (Clemens et al., 2006; Qu et al., 2006, 2007), where patients suffer from an uncontrolled desire to move their limbs associated with modulation of D_3_ receptors in the spinal cord (Maitra et al., 1993). Further clues to the role of the A11 in motor control will require selective activation and inactivation of spinally projecting axons to differentiate direct from indirect actions on spinal cord functions. Given the robust effects of dopamine on modulation of spinal circuits (Sharples et al., 2014) this would be an interesting target to investigate. A11 forms one of many nuclei in the diencephalon and midbrain that have motor effects and connect with nuclei in the brainstem and spinal cord (Kim et al., 2017; Brownstone and Chopek, 2018; Gatto and Goulding, 2018; Sharma et al., 2018). A more complete understanding of their role will require parallel recording and stimulation of these distinct regions during complex motor behavior.

## Author Contributions

KK performed the experiments, analyzed the data, and wrote and edited the paper. CJ-X participated in performing experiments, analyzed data, edited the paper, and participated in experimental design. SS participated in performing experiments, edited the paper, and participated in experimental design. TF edited the paper, analyzed the data, and participated in performing experiments. LY, SEAE, and CHTK performed experiments, analyzed data, and edited the paper. JB participated in experiment design and edited the paper. PW conceived the experiments, supervised the research, and edited and wrote the paper.

## Conflict of Interest Statement

The authors declare that the research was conducted in the absence of any commercial or financial relationships that could be construed as a potential conflict of interest.

## Supplementary Material

VIDEO 1 Photostimulation of A11^ChR2^ mouse during the open field test.

## References

[B1] Abdallah K. Monconduit L. Artola A. Luccarini P. Dallel R. (2015). GABAAergic inhibition or dopamine denervation of the A11 hypothalamic nucleus induces trigeminal analgesia. *Pain* 156 644–655. 10.1097/j.pain.0000000000000091 25790455

[B2] Abrahamson E. E. Moore R. Y. (2001). The posterior hypothalamic area: chemoarchitecture and afferent connections. *Brain Res.* 889 1–22. 10.1016/S0006-8993(00)03015-8 11166682

[B3] Barraud Q. Obeid I. Aubert I. Barrière G. Contamin H. Mcguire S. (2010). Neuroanatomical study of the A11 diencephalospinal pathway in the non-human primate. *PLoS One* 5:e13306. 10.1371/journal.pone.0013306 20967255 PMC2954154

[B4] Barrière G. Mellen N. Cazalets J.-R. (2004). Neuromodulation of the locomotor network by dopamine in the isolated spinal cord of newborn rat. *Eur. J. Neurosci.* 19 1325–1335. 10.1111/j.1460-9568.2004.03210.x 15016090

[B5] Björklund A. Skagerberg G. (1979). Evidence for a major spinal cord projection from the diencephalic A11 dopamine cell group in the rat using transmitter-specific fluorescent retrograde tracing. *Brain Res.* 177 170–175. 10.1016/0006-8993(79)90927-2 497819

[B6] Brownstone R. M. Chopek J. W. (2018). Reticulospinal systems for tuning motor commands. *Front. Neural Circuits* 12:30. 10.3389/fncir.2018.00030 29720934 PMC5915564

[B7] Bunzow J. R. Sonders M. S. Arttamangkul S. Harrison L. M. Zhang G. Quigley D. I. (2001). Amphetamine, 3,4-methylenedioxymethamphetamine, lysergic acid diethylamide, and metabolites of the catecholamine neurotransmitters are agonists of a rat trace amine receptor. *Mol. Pharmacol.* 60 1181–1188. 10.1124/mol.60.6.1181 11723224

[B8] Bustin S. A. Benes V. Garson J. A. Hellemans J. Huggett J. Kubista M. (2009). The MIQE guidelines: minimum information for publication of quantitative real-time PCR experiments. *Clin. Chem.* 55 611–622. 10.1373/clinchem.2008.112797 19246619

[B9] Charbit A. R. Akerman S. Holland P. R. Goadsby P. J. (2009). Neurons of the dopaminergic/calcitonin gene-related peptide A11 cell group modulate neuronal firing in the trigeminocervical complex: an electrophysiological and immunohistochemical study. *J. Neurosci.* 29 12532–12541. 10.1523/JNEUROSCI.2887-09.2009 19812328 PMC6665099

[B10] Christie K. J. Whelan P. J. (2005). Monoaminergic establishment of rostrocaudal gradients of rhythmicity in the neonatal mouse spinal cord. *J. Neurophysiol.* 94 1554–1564. 10.1152/jn.00299.2005 15829596

[B11] Clemens S. Belin-Rauscent A. Simmers J. Combes D. (2012). Opposing modulatory effects of D1- and D2-like receptor activation on a spinal central pattern generator. *J. Neurophysiol.* 107 2250–2259. 10.1152/jn.00366.2011 22262823

[B12] Clemens S. Hochman S. (2004). Conversion of the modulatory actions of dopamine on spinal reflexes from depression to facilitation in D3 receptor knock-out mice. *J. Neurosci.* 24 11337–11345. 10.1523/JNEUROSCI.3698-04.2004 15601940 PMC2731231

[B13] Clemens S. Rye D. Hochman S. (2006). Restless legs syndrome: revisiting the dopamine hypothesis from the spinal cord perspective. *Neurology* 67 125–130. 10.1212/01.wnl.0000223316.53428.c9 16832090

[B14] Commissiong J. W. Gentleman S. Neff N. H. (1979). Spinal cord dopaminergic neurons: evidence for an uncrossed nigrospinal pathway. *Neuropharmacology* 18 565–568. 10.1016/0028-3908(79)90102-3 481709

[B15] Dahlström A. Fuxe K. (1964). Evidence for the existence of monoamine-containing neurons in the central nervous system. I. Demonstration of monoamines in the cell bodies of brain stem neurons. *Acta Physiol. Scand. Suppl.* 232 1–55. 14229500

[B16] Fleetwood-Walker S. M. Hope P. J. Mitchell R. (1988). Antinociceptive actions of descending dopaminergic tracts on cat and rat dorsal horn somatosensory neurones. *J. Physiol.* 399 335–348. 10.1113/jphysiol.1988.sp017084 2841456 PMC1191668

[B17] Gatto G. Goulding M. (2018). Locomotion control: brainstem circuits satisfy the need for speed. *Curr. Biol.* 28 R256–R259. 10.1016/j.cub.2018.01.068 29558639 PMC5942195

[B18] Gerin C. Privat A. (1998). Direct evidence for the link between monoaminergic descending pathways and motor activity: II. A study with microdialysis probes implanted in the ventral horn of the spinal cord. *Brain Res.* 794 169–173. 10.1016/S0006-8993(98)00278-9 9630613

[B19] Gordon I. T. Whelan P. J. (2006). Monoaminergic control of cauda-equina-evoked locomotion in the neonatal mouse spinal cord. *J. Neurophysiol.* 96 3122–3129. 10.1152/jn.00606.2006 16956991

[B20] Gozal E. A. O’Neill B. E. Sawchuk M. A. Zhu H. Halder M. Chou C.-C. (2014). Anatomical and functional evidence for trace amines as unique modulators of locomotor function in the mammalian spinal cord. *Front. Neural Circuits* 8:134. 10.3389/fncir.2014.00134 25426030 PMC4224135

[B21] Han P. Nakanishi S. T. Tran M. A. Whelan P. J. (2007). Dopaminergic modulation of spinal neuronal excitability. *J. Neurosci.* 27 13192–13204. 10.1523/JNEUROSCI.1279-07.2007 18045913 PMC6673410

[B22] Han P. Whelan P. J. (2009). Modulation of AMPA currents by D(1)-like but not D(2)-like receptors in spinal motoneurons. *Neuroscience* 158 1699–1707. 10.1016/j.neuroscience.2008.11.040 19110039

[B23] Hochman S. (2015). Metabolic recruitment of spinal locomotion: intracellular neuromodulation by trace amines and their receptors. *Neural Regen. Res.* 10 1940–1942. 10.4103/1673-5374.169625 26889178 PMC4730814

[B24] Humphreys J. Whelan P. (2011). Dopamine exerts activation dependent modulation of spinal locomotor circuits in the mouse. *J. Neurophysiol.* 108 3370–3381. 10.1152/jn.00482.2012 22993259

[B25] Humphreys J. M. Whelan P. J. (2012). Dopamine exerts activation-dependent modulation of spinal locomotor circuits in the neonatal mouse. *J. Neurophysiol.* 108 3370–3381. 10.1152/jn.00482.2012 22993259

[B26] Jaeger C. B. Teitelman G. Joh T. H. Albert V. R. Park D. H. Reis D. J. (1983). Some neurons of the rat central nervous system contain aromatic-L-amino-acid decarboxylase but not monoamines. *Science* 219 1233–1235. 10.1126/science.6131537 6131537

[B27] Jiang Z. Carlin K. P. Brownstone R. M. (1999). An in vitro functionally mature mouse spinal cord preparation for the study of spinal motor networks. *Brain Res.* 816 493–499. 10.1016/S0006-8993(98)01199-8 9878874

[B28] Jordan L. M. Schmidt B. J. (2002). Propriospinal neurons involved in the control of locomotion: potential targets for repair strategies? *Prog. Brain Res.* 137 125–139. 10.1016/S0079-6123(02)37012-2 12440364

[B29] Kiehn O. Kjaerulff O. (1996). Spatiotemporal characteristics of 5-HT and dopamine-induced rhythmic hindlimb activity in the in vitro neonatal rat. *J. Neurophysiol.* 75 1472–1482. 10.1152/jn.1996.75.4.1472 8727391

[B30] Kiehn O. Sillar K. T. Kjaerulff O. McDearmid J. R. (1999). Effects of noradrenaline on locomotor rhythm–generating networks in the isolated neonatal rat spinal cord. *J. Neurophysiol.* 82 741–746. 10.1152/jn.1999.82.2.741 10444672

[B31] Kim L. H. Sharma S. Sharples S. A. Mayr K. A. Kwok C. H. T. Whelan P. J. (2017). Integration of descending command systems for the generation of context-specific locomotor behaviors. *Front. Neurosci.* 11:581. 10.3389/fnins.2017.00581 29093660 PMC5651258

[B32] Koblinger K. Füzesi T. Ejdrygiewicz J. Krajacic A. Bains J. S. Whelan P. J. (2014). Characterization of a11 neurons projecting to the spinal cord of mice. *PLoS One* 9:e109636. 10.1371/journal.pone.0109636 25343491 PMC4208762

[B33] Koblinger K. Jean-Xavier C. Ejdrygiewicz J. Fuzesi T. Bains J. S. Whelan P. J. (2015). “A11 optogenetic stimulation elicits locomotor activity in mice,” in *Conference Society for Neuroscience*, Chicago, IL.

[B34] Lambert A. M. Bonkowsky J. L. Masino M. A. (2012). The conserved dopaminergic diencephalospinal tract mediates vertebrate locomotor development in zebrafish larvae. *J. Neurosci.* 32 13488–13500. 10.1523/JNEUROSCI.1638-12.2012 23015438 PMC3481997

[B35] Lammel S. Steinberg E. E. Földy C. Wall N. R. Beier K. Luo L. (2015). Diversity of transgenic mouse models for selective targeting of midbrain dopamine neurons. *Neuron* 85 429–438. 10.1016/j.neuron.2014.12.036 25611513 PMC5037114

[B36] Lapointe N. P. Rouleau P. Ung R.-V. Guertin P. A. (2009). Specific role of dopamine D1 receptors in spinal network activation and rhythmic movement induction in vertebrates. *J. Physiol.* 587 1499–1511. 10.1113/jphysiol.2008.166314 19204052 PMC2678221

[B37] Liu J. Jordan L. M. (2005). Stimulation of the parapyramidal region of the neonatal rat brain stem produces locomotor-like activity involving spinal 5-HT7 and 5-HT2A receptors. *J. Neurophysiol.* 94 1392–1404. 10.1152/jn.00136.2005 15872068

[B38] Madriaga M. A. McPhee L. C. Chersa T. Christie K. J. Whelan P. J. (2004). Modulation of locomotor activity by multiple 5-HT and dopaminergic receptor subtypes in the neonatal mouse spinal cord. *J. Neurophysiol.* 92 1566–1576. 10.1152/jn.01181.2003 15163678

[B39] Maitra K. K. Seth P. Thewissen M. Ross H. G. Ganguly D. K. (1993). Dopaminergic influence on the excitability of antidromically activated Renshaw cells in the lumbar spinal cord of the rat. *Acta Physiol. Scand.* 148 101–107. 10.1111/j.1748-1716.1993.tb09538.x 8352022

[B40] McCrea A. E. Stehouwer D. J. van Hartesveldt C. (1997). Dopamine D1 and D2 antagonists block L-DOPA-induced air-stepping in decerebrate neonatal rats. *Brain Res. Dev. Brain Res.* 100 130–132. 10.1016/S0165-3806(97)00027-8 9174256

[B41] Megat S. Shiers S. Moy J. K. Barragan-Iglesias P. Pradhan G. Seal R. P. (2018). A critical role for dopamine d5 receptors in pain chronicity in male mice. *J. Neurosci.* 38 379–397. 10.1523/JNEUROSCI.2110-17.2017 29167404 PMC5761615

[B42] Meng D. Li H.-Q. Deisseroth K. Leutgeb S. Spitzer N. C. (2018). Neuronal activity regulates neurotransmitter switching in the adult brain following light-induced stress. *Proc. Natl. Acad. Sci. U.S.A.* 115 5064–5071. 10.1073/pnas.1801598115 29686073 PMC5960321

[B43] Millan M. J. (2002). Descending control of pain. *Prog. Neurobiol.* 66 355–474. 10.1016/S0301-0082(02)00009-6 12034378

[B44] Mori S. Sakamoto T. Ohta Y. Takakusaki K. Matsuyama K. (1989). Site-specific postural and locomotor changes evoked in awake, freely moving intact cats by stimulating the brainstem. *Brain Res.* 505 66–74. 10.1016/0006-8993(89)90116-9 2611678

[B45] Okura M. Fujiki N. Kita I. Honda K. Yoshida Y. Mignot E. (2004). The roles of midbrain and diencephalic dopamine cell groups in the regulation of cataplexy in narcoleptic Dobermans. *Neurobiol. Dis.* 16 274–282. 10.1016/j.nbd.2004.02.008 15207284

[B46] Pappas S. S. Behrouz B. Janis K. L. Goudreau J. L. Lookingland K. J. (2008). Lack of D2 receptor mediated regulation of dopamine synthesis in A11 diencephalospinal neurons in male and female mice. *Brain Res.* 1214 1–10. 10.1016/j.brainres.2008.03.010 18462709

[B47] Pappas S. S. Tiernan C. T. Behrouz B. Jordan C. L. Breedlove S. M. Goudreau J. L. (2010). Neonatal androgen-dependent sex differences in lumbar spinal cord dopamine concentrations and the number of A11 diencephalospinal dopamine neurons. *J. Comp. Neurol.* 518 2423–2436. 10.1002/cne.22340 20503420 PMC3884812

[B48] Peyron C. Luppi P. H. Kitahama K. Fort P. Hermann D. M. Jouvet M. (1995). Origin of the dopaminergic innervation of the rat dorsal raphe nucleus. *Neuroreport* 6 2527–2531. 10.1097/00001756-199512150-00019 8741755

[B49] Picton L. D. Nascimento F. Broadhead M. J. Sillar K. T. Miles G. B. (2017). Sodium pumps mediate activity-dependent changes in mammalian motor networks. *J. Neurosci.* 37 906–921. 10.1523/JNEUROSCI.2005-16.2016 28123025 PMC5296784

[B50] Qu S. Le W. Zhang X. Xie W. Zhang A. Ondo W. G. (2007). Locomotion is increased in a11-lesioned mice with iron deprivation: a possible animal model for restless legs syndrome. *J. Neuropathol. Exp. Neurol.* 66 383–388. 10.1097/nen.0b013e3180517b5f 17483695

[B51] Qu S. Ondo W. G. Zhang X. Xie W. J. Pan T. H. Le W. D. (2006). Projections of diencephalic dopamine neurons into the spinal cord in mice. *Exp. Brain Res.* 168 152–156. 10.1007/s00221-005-0075-1 16044299

[B52] Romanov R. A. Zeisel A. Bakker J. Girach F. Hellysaz A. Tomer R. (2017). Molecular interrogation of hypothalamic organization reveals distinct dopamine neuronal subtypes. *Nat. Neurosci.* 20 176–188. 10.1038/nn.4462 27991900 PMC7615022

[B53] Ryu S. Mahler J. Acampora D. Holzschuh J. Erhardt S. Omodei D. (2007). Orthopedia homeodomain protein is essential for diencephalic dopaminergic neuron development. *Curr. Biol.* 17 873–880. 10.1016/j.cub.2007.04.003 17481897

[B54] Schmidt B. J. Jordan L. M. (2000). The role of serotonin in reflex modulation and locomotor rhythm production in the mammalian spinal cord. *Brain Res. Bull.* 53 689–710. 10.1016/S0361-9230(00)00402-0 11165804

[B55] Seth P. Gajendiran M. Maitra K. K. Ross H. G. Ganguly D. K. (1993). Evidence for D1 dopamine receptor-mediated modulation of the synaptic transmission from motor axon collaterals to Renshaw cells in the rat spinal cord. *Neurosci. Lett.* 158 217–220. 10.1016/0304-3940(93)90268-P 8233099

[B56] Sharma S. Kim L. H. Mayr K. A. Elliott D. A. Whelan P. J. (2018). Parallel descending dopaminergic connectivity of A13 cells to the brainstem locomotor centers. *Sci. Rep.* 8:7972. 10.1038/s41598-018-25908-5 29789702 PMC5964077

[B57] Sharples S. A. Humphreys J. M. Jensen A. M. Dhoopar S. Delaloye N. Clemens S. (2015). Dopaminergic modulation of locomotor network activity in the neonatal mouse spinal cord. *J. Neurophysiol.* 113 2500–2510. 10.1152/jn.00849.2014 25652925 PMC4416552

[B58] Sharples S. A. Koblinger K. Humphreys J. M. Whelan P. J. (2014). Dopamine: a parallel pathway for the modulation of spinal locomotor networks. *Front. Neural Circuits* 8:55. 10.3389/fncir.2014.00055 24982614 PMC4059167

[B59] Sharples S. A. Whelan P. J. (2017). Modulation of rhythmic activity in mammalian spinal networks is dependent on excitability state. *eNeuro* 4:ENEURO.0368-16.2017. 10.1523/ENEURO.0368-16.2017 28144626 PMC5272924

[B60] Shik M. L. Orlovsky G. N. (1976). Neurophysiology of locomotor automatism. *Physiol. Rev.* 56 465–501. 10.1152/physrev.1976.56.3.465 778867

[B61] Sickles A. E. Stehouwer D. J. van Hartesveldt C. (1992). Dopamine D1 and D2 antagonists block L-dopa-elicited air-stepping in neonatal rats. *Brain Res. Dev. Brain Res.* 68 17–22. 10.1016/0165-3806(92)90243-P 1387836

[B62] Sinnamon H. M. Ginzburg R. N. Kurose G. A. (1987). Midbrain stimulation in the anesthetized rat: direct locomotor effects and modulation of locomotion produced by hypothalamic stimulation. *Neuroscience* 20 695–707. 10.1016/0306-4522(87)90120-5 3587613

[B63] Sinnamon H. M. Lee S. H. Adams D. B. Stopford C. K. (1984). Locomotor stepping elicited by electrical stimulation of the lateral hypothalamus requires an ipsilateral descending pathway. *Physiol. Behav.* 33 209–215. 10.1016/0031-9384(84)90101-X 6334326

[B64] Sinnamon H. M. Stopford C. K. (1987). Locomotion elicited by lateral hypothalamic stimulation in the anesthetized rat does not require the dorsal midbrain. *Brain Res.* 402 78–86. 10.1016/0006-8993(87)91049-3 3828790

[B65] Skagerberg G. Lindvall O. (1985). Organization of diencephalic dopamine neurones projecting to the spinal cord in the rat. *Brain Res.* 342 340–351. 10.1016/0006-8993(85)91134-5 4041835

[B66] Sławińska U. Kasicki S. (1995). Theta-like rhythm in depth EEG activity of hypothalamic areas during spontaneous or electrically induced locomotion in the rat. *Brain Res.* 678 117–126. 10.1016/0006-8993(95)00174-O 7620881

[B67] Sławińska U. Miazga K. Jordan L. M. (2014). The role of serotonin in the control of locomotor movements and strategies for restoring locomotion after spinal cord injury. *Acta Neurobiol. Exp.* 74 172–187. 10.55782/ane-2014-1983 24993627

[B68] Stuber G. D. Wise R. A. (2016). Lateral hypothalamic circuits for feeding and reward. *Nat. Neurosci.* 19 198–205. 10.1038/nn.4220 26814589 PMC4927193

[B69] Takada M. (1990). The A11 catecholamine cell group: another origin of the dopaminergic innervation of the amygdala. *Neurosci. Lett.* 118 132–135. 10.1016/0304-3940(90)90266-C 1979671

[B70] Takada M. (1993). Widespread dopaminergic projections of the subparafascicular thalamic nucleus in the rat. *Brain Res. Bull.* 32 301–309. 10.1016/0361-9230(93)90191-D 8104090

[B71] Takada M. Li Z. K. Hattori T. (1988). Single thalamic dopaminergic neurons project to both the neocortex and spinal cord. *Brain Res.* 455 346–352. 10.1016/0006-8993(88)90093-5 2900059

[B72] Tamae A. Nakatsuka T. Koga K. Kato G. Furue H. Katafuchi T. (2005). Direct inhibition of substantia gelatinosa neurones in the rat spinal cord by activation of dopamine D2-like receptors. *J. Physiol.* 568 243–253. 10.1113/jphysiol.2005.091843 15975975 PMC1474768

[B73] Taniguchi W. Nakatsuka T. Miyazaki N. Yamada H. Takeda D. Fujita T. (2011). In vivo patch-clamp analysis of dopaminergic antinociceptive actions on substantia gelatinosa neurons in the spinal cord. *Pain* 152 95–105. 10.1016/j.pain.2010.09.034 21050660

[B74] Ugrumov M. V. (2009). Non-dopaminergic neurons partly expressing dopaminergic phenotype: distribution in the brain, development and functional significance. *J. Chem. Neuroanat.* 38 241–256. 10.1016/j.jchemneu.2009.08.004 19698780

[B75] Weihe E. Depboylu C. Schütz B. Schäfer M. K.-H. Eiden L. E. (2006). Three types of tyrosine hydroxylase-positive CNS neurons distinguished by dopa decarboxylase and VMAT2 co-expression. *Cell. Mol. Neurobiol.* 26 659–678. 10.1007/s10571-006-9053-9 16741673 PMC4183211

[B76] Weil-Fugazza J. Godefroy F. (1993). Dorsal and ventral dopaminergic innervation of the spinal cord: functional implications. *Brain Res. Bull.* 30 319–324. 10.1016/0361-9230(93)90259-E 8457880

[B77] Whelan P. Bonnot A. O’Donovan M. J. (2000). Properties of rhythmic activity generated by the isolated spinal cord of the neonatal mouse. *J. Neurophysiol.* 84 2821–2833. 10.1152/jn.2000.84.6.2821 11110812

[B78] Whelan P. J. (2017). *Uncovering Parallel Pathways for Movement Control. Program No. 267.06. 2017 Neuroscience Meeting Planner.* Washington, DC: Society for Neuroscience.

[B79] Yip S. H. York J. Hyland B. Bunn S. J. Grattan D. R. (2017). Incomplete concordance of dopamine transporter Cre (DATIREScre)-mediated recombination and tyrosine hydroxylase immunoreactivity in the mouse forebrain. *J. Chem. Neuroanat.* 90 40–48. 10.1016/j.jchemneu.2017.12.002 29217488

[B80] Yoshida M. Tanaka M. (1988). Existence of new dopaminergic terminal plexus in the rat spinal cord: assessment by immunohistochemistry using anti-dopamine serum. *Neurosci. Lett.* 94 5–9. 10.1016/0304-3940(88)90261-3 3071747

[B81] Young C. K. Koke S. J. Kiss Z. H. Bland B. H. (2009). Deep brain stimulation of the posterior hypothalamic nucleus reverses akinesia in bilaterally 6-hydroxydopamine-lesioned rats. *Neuroscience* 162 1–4. 10.1016/j.neuroscience.2009.04.053 19401216

[B82] Zhu H. Clemens S. Sawchuk M. A. Hochman S. (2007). Expression and distribution of all dopamine receptor subtypes (D(1)-D(5)) in the mouse lumbar spinal cord: a real-time polymerase chain reaction and non-autoradiographic in situ hybridization study. *Neuroscience* 149 885–897. 10.1016/j.neuroscience.2007.07.052 17936519 PMC2185067

